# Coral reefs: The good and not so good news with future bright and dark spots for coral reefs through climate change

**DOI:** 10.1111/gcb.16271

**Published:** 2022-06-07

**Authors:** Michelle J. Devlin

**Affiliations:** ^1^ CEFAS Lowestoft UK

**Keywords:** climate change, coral reefs, pollution, recovery, resilience

## Abstract

COMMENTARY ON Present and future bright and dark spots for coral reefs through climate change.

This is a commentary on Sully et al., 2022, https://doi.org/10.1111/gcb.16083

Coral reefs are one of the most threatened habitats on earth with up to 67% of historical global extent now lost and much of the remaining intact coral reef systems facing unprecedented risk from climate change, overfishing and pollution (Eakin et al., 2010; Hughes et al., 2018; MacNeil et al., 2019; Ortiz et al., 2018; Wolff et al., 2018). Coral reefs are vital for our oceans, supporting many different types of marine species, but also providing livelihoods for over 600 million people, and coastal protection for millions more. Climate change, through increasing temperature and carbon dioxide, impacts all coral reefs, though the responses are variable across coral reefs, with highest global losses in the Eastern Pacific, South Asia and the Great Barrier Reef. Local and regional pressures are also driving negative impacts, through overfishing and improper fishing practices, coastal development, sedimentation, land‐based sources of pollution and marine pollution (Brodie et al., 2010, 2012; Devlin et al., 2012; Duran et al., 2018; Dutra et al., 2021).

The outlook is and continues to be gloomy for many of our coral reefs. Work from Hughes et al. (2003) and Pandolfi et al. (2003) had identified this crisis in the early 2000s with projected increases in carbon dioxide and temperature over the next 50 years driving unsuitable conditions for coral reefs, which, in turn, were predicted to lead to regime shifts in many of our coral reef communities. This outcome has been confirmed in a depressing number of science publications, with catastrophic shifts in coral reef cover (Hughes et al., 2017, 2018; Sheppard et al., 2010), synergistic impacts of warming and acidification (Dove et al., 2020) and reductions in ecosystem services (Eddy et al., 2021).

However, a small but significant shift being measured in coral reef trajectories may mean that coral reefs can survive these anthropogenic pressures if given enough time to recover and ongoing reduction in many of the local, direct pressures. While the recent Global Coral Reef Monitoring Network report (Souter et al., 2021) does document substantial declines in coral reef cover from a 2002 baseline, the period in the early 2000s showed good recovery across many coral reefs. While the global 2010 and 2015–2017 bleaching events reversed this recovery, there have been small increases in coral cover in the last 2 years, highlighting once again that coral reefs are robust and can recover, given enough time and reduced localised pressures to provide favorable conditions for the faster growing corals. Pressures such as overfishing and marine pollution can be mitigated through strong local and regional actions. Local interventions can have far‐reaching impacts with many of the actions to improve water quality available at community and national levels (Brodie et al., 2019; Devlin et al., 2021; Waterhouse et al., 2017). Recent studies have also highlighted the potential for coral reefs to adapt to increasing temperatures after bleaching events, with high post‐disturbance survivorship of recruits (Adjeroud et al., 2018; Cannon et al., 2021; Hoegh‐Guldberg et al., 2018; Mcleod et al., 2019). This recovery can also be supported through targeted management of coral reefs that have higher tolerances or are important in supply coral larvae to surrounding reefs (Mumby et al., 2021). Smaller scale interventions can include local community programmes focusing on coral reef restoration (Bayraktarov et al., 2019), local management and monitoring (Johnson et al., 2020) and community‐led adaptations (Dutra et al., 2021; White & Vogt, 2000). Whilst coral reefs change and adapt in real time to a multitude of anthropogenic pressures, there is still hope that, with strong global interventions for climate change and a clear set of interventions for marine pollution, overfishing and coastal development, we can support and protect our coral reefs.

Sully et al. (2022) add to this vital narrative, with a study on future bright and dark spots for coral reefs through climate change. They analysed 7714 worldwide surveys from 1997 to 2018 along with 14 environmental and temperature metrics in a hierarchical Bayesian model to identify conditions that contribute to present‐day coral cover. While the projections for RCP8.5 predict catastrophic relative decreases in coral cover, this is variable, with bright spots identified for Indonesia, Malaysia, central Philippines, New Caledonia, Fiji and French Polynesia. The positive correlation of percent coral cover with historical maximum SST may indicate that corals living on reefs historically exposed to relatively high maximum temperatures have suffered less from recent marine heatwaves than corals living elsewhere, supporting the emerging consensus that precondition and local adaptation can support higher survival of corals to high temperature events. The modelling also shows percent coral cover has a negative relationship with tropical cyclone frequency, providing a respite for coral reefs where the severity of cyclones is predicted to decrease, and a worrying future issue for lower latitudes where cyclone severity will increase. Ultimately, this work, as with many others, ends with a strong message of what is in store for coral reefs, biodiversity and the many millions of people who depend on coral reefs if we do not act simultaneously on climate change, marine pollution and overdevelopment. But there is no doubt that some coral reefs are surviving, recovering and adapting, offering glimpses of bright spots, which can be supported through targeted measures on effective local management to support resilience and provide focal sites for conservation. Coral reefs are in crisis, and knowledge of how, when and what we can do in both the short and long term are essential for giving coral reefs the best hope of survival.

## Data Availability

Data sharing is not applicable to this article as no new data were created or analyzed in this study.
